# SNR-Enhanced, Rapid Electrical Conductivity Mapping Using Echo-Shifted MRI

**DOI:** 10.3390/tomography8010031

**Published:** 2022-02-05

**Authors:** Hyunyeol Lee, Jaeseok Park

**Affiliations:** 1 School of Electronics Engineering, Kyungpook National University, Daegu 41566, Korea; 2 Graduate School of Electronic and Electrical Engineering, Kyungpook National University, Daegu 41566, Korea; 3 Department of Biomedical Engineering, Sungkyunkwan University, Suwon 16419, Korea; jaeseokp@skku.edu

**Keywords:** magnetic resonance imaging (MRI), magnetic resonance electrical impedance tomography (MREIT), echo-shifted MRI, steady-state incoherent imaging, electrical conductivity

## Abstract

Magnetic resonance electrical impedance tomography (MREIT) permits high-spatial resolution electrical conductivity mapping of biological tissues, and its quantification accuracy hinges on the signal-to-noise ratio (SNR) of the current-induced magnetic flux density (*B*_z_). The purpose of this work was to achieve *B*_z_ SNR-enhanced rapid conductivity imaging by developing an echo-shifted steady-state incoherent imaging-based MREIT technique. In the proposed pulse sequence, the free-induction-decay signal is shifted in time over multiple imaging slices, and as a result is exposed to a plurality of injecting current pulses before forming an echo. Thus, the proposed multi-slice echo-shifting strategy allows a high SNR for *B*_z_ for a given number of current injections. However, with increasing the time of echo formation, the *B*_z_ SNR will also be compromised by *T*_2_*-related signal loss. Hence, numerical simulations were performed to evaluate the relationship between the echo-shifting and the *B*_z_ SNR, and subsequently to determine the optimal imaging parameters. Experimental studies were conducted to evaluate the effectiveness of the proposed method over conventional spin-echo-based MREIT. Compared with the reference spin-echo MREIT, the proposed echo-shifting-based method improves the efficiency in both data acquisition and current injection while retaining the accuracy of conductivity quantification. The results suggest the feasibility of the proposed MREIT method as a practical means for conductivity mapping.

## 1. Introduction

Electrical conductivity inside the living body is determined by a number of factors, including its underlying cellular structure, ion mobility and concentration, and molecular composition [1,2]. Hence, reliable measurements of electrical conductivity would provide important insights into the physiology of biological tissues in health and disease. In fact, electrical conductivity information has been employed in a range of applications such as EEG and MEG neuronal source localization [3,4,5], quantitative monitoring of neuronal depolarization [6], ion mobility imaging [7,8,9], estimation of current distribution during therapeutic electrical stimulation [10,11,12,13], and evaluation of brain abnormalities [14].

Electrical impedance tomography (EIT) is a non-invasive imaging technique that permits the estimation of the electrical conductivity distribution inside an imaging object [15,16]. In EIT, current injection is applied to the imaging object through multiple surface electrodes, and induced voltages are then measured to reconstruct cross-sectional conductivity maps based on a nonlinear inverse solution. However, the boundary voltage is insensitive to internal conductivity variations, making the inverse problem highly ill-posed and resulting in inaccurate conductivity estimates in regions away from the measurement electrodes [16]. Additionally, it is difficult to achieve high spatial resolution conductivity mapping with a limited number of electrodes [16].

As an alternative, an MRI-based technique, typically referred to as magnetic resonance electrical impedance tomography (MREIT) [17], has been suggested to obtain electrical conductivity maps with sufficiently high spatial resolution and accuracy. In the method, an imaging object is subjected to a series of short current pulses in sync with an MR pulse sequence, and the induced magnetic flux density (**B** = [*B*_x_, *B*_y_, *B*_z_]), which perturbs the local magnetic field, is estimated via phase analysis of acquired MR images. Once all three components of B are measured separately by rotating the object twice inside the MRI scanner, the current density (J) distribution is calculated using Ampere’s law J=∇×B/μ (μ: magnetic permeability), leading to conductivity estimation [18,19]. More recently, a harmonic *B*_z_-based MREIT [20,21] has been introduced to avoid technical difficulties in object rotation in the J-based approaches. Given two sets of *B*_z_ (rather than B) obtained with current injection in two orthogonal directions, the harmonic *B*_z_ method reconstructs conductivity distribution (σ) by exploiting the relationship between $\nabla^{2}B_{z}$ and ∇σ, thus obviating the need for object rotation in MREIT experiments. Despite the advantage, the method may suffer from noise amplifications in the numerical computation of $\nabla^{2}B_{z}$ and thus requires a sufficiently high signal-to-noise ratio (SNR) of *B*_z_.

Previous studies analyzing *B*_z_ SNR have shown that the standard deviation of *B*_z_ is inversely proportional to the SNR of a magnitude image, duration of a current pulse, and further phase sensitivity of an MR pulse sequence to the injection current [22,23]. In this regard, the spin echo (SE) pulse sequence has been widely accepted in MREIT, as it provides a high SNR in magnitude images and allows a long time for current injection (TC) between an excitation RF pulse and signal readout. To further enhance the *B*_z_ SNR without compromising imaging efficiency, multi-echo variants of SE imaging have also been explored [24,25]. Nevertheless, the utility of these SE-based methods has been limited by the impractically long scan times and low phase sensitivity. To address these issues in SE-based MREIT, alternating steady-state free precession (SSFP) MREIT has been suggested [23,26]. Compared with SE MREIT, the method enables rapid conductivity imaging while yielding high phase sensitivity resulting from the nonlinear behavior of SSFP signals in response to alternating current injection. However, in solving the corresponding nonlinear inverse problem, alternating SSFP MREIT requires knowledges of tissue relaxation times and transmit RF inhomogeneities, for which additional measurement scans need to be performed, making the *B*_z_ estimation procedure rather complicated.

In this work, we aimed to overcome the above-mentioned limitations in current MREIT techniques by developing a multi-slice interleaving echo-shifted steady-state incoherent (ESSSI) imaging-based MREIT method so as to achieve current-efficient, high-speed conductivity imaging and direct extraction of *B*_z_ values from acquired-image signals. Free induction decay (FID) signals are shifted in time over multiple imaging slices before forming an echo, thereby being exposed to a multitude of current pulses and accumulating current-induced phases successively, leading to an elevated *B*_z_ SNR without an increase in TC. Number of echo-shifting (NES) values were optimized using numerical simulations. Experimental studies were performed in phantoms to evaluate the effectiveness of the proposed method in comparison to conventional SE MREIT.

## 2. Materials and Methods

### 2.1. Multi-Slice ESSSI Imaging with Current Injection

**Figure 1:**
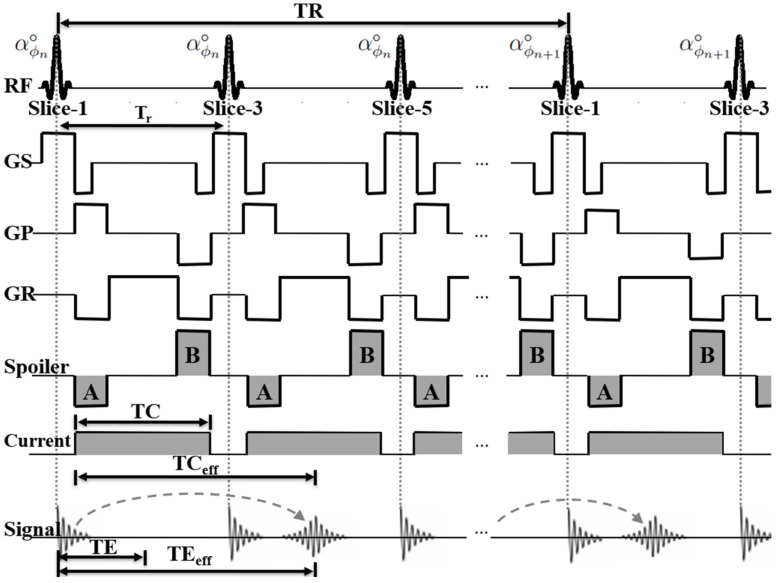
A timing diagram of the proposed multi-slice interleaving echo-shifted MREIT pulse sequence for NES = 1 (GS: slice selection gradient; GP: phase-encoding gradient; GR: frequency-encoding gradient). Note that the FID signals are shifted in time over multiple imaging slices to form an echo at TE_eff_ rather than TE. Note also that current pulses with a constant amplitude are applied during an interval between two neighboring RF pulses. Thus, the effective time of current injection (TC_eff_) that each echo signal experiences is longer than the TC.

A timing diagram of the proposed multi-slice interleaving ESSSI imaging method with current injection is shown in Figure 1. Equidistant, spatially selective excitation RF pulses with constant flip angles (α◦) are successively applied to corresponding, interleaved imaging slices. To achieve maximal incoherence between isochromats at the end of each time of repetition (TR), RF spoiling is applied with the quadratic phase cycling scheme [27]:

(1) $$\phi_{n+1}-\phi_{n}=n\psi ,\;\;\;n=1,\;2,\;3,\ldots$$

where ϕ is the RF phase, *n* is the RF index for each slice, and ψ is the RF phase increment, typically set to 117° so as to produce a signal intensity comparable to that achieved by ideal spoiling [27]. The time integral of gradient pulses between any two neighboring RF pulses is kept identical in all three directions (x, y, z) to avoid artifacts otherwise resulting from temporally varying spin dephasing. Further, a pair of spoiler gradients is inserted before and after each signal readout (see below for details) while a short, unipolar current pulse is applied between each pair of RF pulses. With the above configuration, a current-encoded incoherent steady state is established by increasing the number of RF pulses.

In the present pulse sequence, an FID signal produced in an imaging slice is shifted in time to the following blocks led by RF pulses for other imaging slices (Figure 1), forming an echo at an effective time of echo (TE_eff_):

(2) $$TE_{eff}=TE+NES\cdot T_{r}$$

where T_r_ is the time interval between two neighboring RF pulses. With this echo-shifting scheme, each FID signal is exposed to more than one current pulse without an increase in TR, while each current pulse is effectively shared by NES slices. Hence, compared with non-echo-shifting multi-slice SSI imaging, the proposed method enables rapid and current-efficient conductivity imaging. A detailed analysis of the effect of echo-shifting on the *B*_z_ SNR is provided in the next section.

To achieve echo-shifting over multiple slices, two spoiler gradient pulses (A and B in Figure 1) are positioned before and after each signal readout with the following design criteria:

(3) $$\left\{\begin{array}{c} \gamma \left|M_{B}\right|\Delta x\geq 2\pi ,\;\;\;\;\;\;\;\;\;NES=0\\ \gamma \left|\left(m+1\right)M_{A}+mM_{B}\right|\Delta x\geq 2\pi ,\;\;\;\;NES\geq 1 \end{array}\right.$$

Here, γ is the gyromagnetic ratio; M_A_ and M_B_ are the zeroth moments of the spoilers A and B, respectively; Δ*x* is the voxel size; and *m* is the echo-shifting index ranging from 0 to NES-1. Equation (3) ensures that for any NES value, all FID signals experience spoiler-induced spin dephasing over 2π within each voxel so as to avoid their interferences within the window of signal readouts, thereby preventing resultant image artifacts. Given the above considerations, the minimum size of each spoiler gradient is calculated by:

(4) $$M_{A}=\left\{\begin{array}{c} 0,\;\;\;\;\;\;\;\;\;NES=0\\ \frac{2\pi \cdot NES}{\gamma \Delta x},\;\;\;\;NES\geq 1 \end{array}\right.$$

and:

(5) $$M_{B}=\left\{\begin{array}{c} \frac{2\pi }{\gamma \Delta x},\;\;\;\;\;\;\;\;\;NES=0\\ -\left(1+\frac{1}{NES}\right)\cdot M_{A},\;\;\;\;NES\geq 1 \end{array}\right.$$

The total amount of gradient-induced spin dephasing during each TR is then given by 2π times the number of excitation slices, which is sufficiently large for effective RF spoiling [27,28].

### 2.2. B_z_ Estimation and SNR Analysis

With the integration of injection currents into the ESSSI pulse sequence, the effective TC (TC_eff_), the time for which an FID signal encodes the current-induced magnetic field (*B*_z_), is defined as:

(6) $$TC_{eff}=TE+NES\cdot TC$$

As a result, the phase of a forming echo at TE_eff_ is given by:

(7) $$\phi_{b}+\phi_{c}=\gamma (\Delta B_{0}TE_{eff}+B_{z}TC_{eff})$$

while its magnitude signal is determined by a transverse relaxation time constant, $T_{2,eff}^{*}$, expressed as:

(8) $$\frac{1}{T_{2,eff}^{*}}=\frac{1}{T_{2}}+\gamma (\Delta B_{0}+B_{z})=\frac{1}{T_{2}^{*}}+\gamma B_{z}$$

Here, $\phi_{b}$ and $\phi_{c}$ are phases due to the background magnetic field ($\Delta B_{0}$) and *B*_z_, respectively. In Equation (8), it is assumed that $\Delta B_{0}$ lends itself to a Lorentzian spectral distribution while *B*_z_ is piecewise constant. 

To selectively extract $\phi_{c}$ values from acquired signals, in this work two separate ESSSI MREIT scans were performed, in which current pulses with positive (+I) and negative (−I) polarities were applied, respectively, with the same amplitudes and durations. The corresponding steady-state signals in a voxel, S^+^ and S^−^, can be written as:

(9) $$\begin{array}{c} S^{\pm }=S_{M}\cdot e^{j\left(\phi_{b}\pm \phi_{c}\right)}\\ S_{M}=\frac{M_{0}\sin\alpha \left(1-E_{1}\right)}{1-E_{1}\cos\alpha }e^{-TE_{eff}/T_{2,eff}^{*}} \end{array}$$

where S_M_ is the signal magnitude, M_0_ is the magnetization in the thermal equilibrium state, and $E_{1}=e^{-TR/T_{1}}$. The current-induced *B*_z_ value in each voxel is then calculated by:

(10) $$B_{z}\left(r\right)=\frac{1}{2\gamma TC_{eff}}\arg\left(\frac{S^{+}\left(r\right)}{S^{-}\left(r\right)}\right)$$

Here, r is the voxel position and arg(·) is the operator that yields the phase of its argument.

Noise analysis in MREIT [22,23] reveals that the standard deviation of *B*_z_ ($SD_{B_{z}}$) can be represented by ${\left(\sqrt{2}\gamma \cdot TC\cdot \Upsilon_{M}\cdot \xi \right)}^{-1}$, where $\Upsilon_{M}$ is the SNR of magnitude image and ξ is the phase sensitivity of an MR pulse sequence to the injection current. In ESSSI MREIT, both TC and $\Upsilon_{M}$ are functions of NES (Equations (6) and (9)), while the image phase accumulates linearly with ξ=1. Hence, $SD_{B_{z}}$ in the proposed method can be written as:

(11) $$SD_{B_{z}}={\left(\sqrt{2}\gamma \cdot TC_{eff}\cdot \Upsilon_{M,ESSSI}\right)}^{-1}$$

The above equations imply that the two factors, TC_eff_ and $\Upsilon_{M,ESSSI}$, conflict with each other in maximizing *B*_z_ SNR. Furthermore, with an Ernst flip angle employed, the signal intensity (S_M_ in Equation (9)) is determined by TE_eff_ and TR. Given these considerations, for a given number of imaging slices, the two imaging parameters, NES and T_r_, are critical determinants of *B*_z_ SNR and are optimized using numerical simulations in the next section.

### 2.3. Numerical Simulations

Numerical simulations were performed in the proposed ESSSI MREIT process to determine an optimal combination of NES and T_r_ that yields a maximal *B*_z_ SNR for a range of tissue relaxation times. To this end, the gain of *B*_z_ SNR (η) achievable with the echo-shifting scheme relative to non-echo-shifting (i.e., NES = 0), was defined as:

(12) $$\eta =\frac{SD_{B_{z}}\left(NES=0\right)}{SD_{B_{z}}\left(NES\neq 0\right)}$$

With increasing NES values from 0 to 7 and varying T_r_ times from 5 ms to 15 ms, contour plots of η were generated for three different tissues with $T_{2}^{*}$ values of 40, 70, and 100 ms, respectively. In the ESSSI pulse sequence, as NES increases the size of the two spoiler gradients needs to be enlarged, thereby increasing the minimum possible T_r_ under the limit of a maximum gradient amplitude (G_max_). This systematic lower bound of T_r_ was calculated for each NES value and was indicated in the contour plots. The simulation parameters were: TE = 5 ms, number of slices = 8, current-induced *B*_z_ = 20 nT, RF pulse duration = 1 ms, and T_1_/M_0_ = 500 ms/1.0. Both TR and TC were adjusted with T_r_. Δ*x* = 1.5 mm and G_max_ = 28 mT/m being assumed.

To evaluate the proposed method’s performance over the conventional SE MREIT approach in terms of *B_z_* SNR per unit scan time, simulations were performed using the following equation:

(13) $$\xi =\frac{SNR_{Bz,ESSSI}/TR_{ESSSI}}{SNR_{Bz,SE}/TR_{SE}}=\frac{SD_{Bz,SE}\cdot TR_{SE}}{SD_{Bz,ESSSI}\cdot TR_{ESSSI}}$$

where SNR_Bz_ is the SNR of the *B_z_* values calculated in each imaging method. Hence, ξ represents the *B_z_* SNR efficiency of the proposed ESSSI technique relative to SE imaging. Here, $SD_{Bz,ESSSI}$ was obtained using Equation (11), while $SD_{Bz,SE}$ was derived using ${\left(\sqrt{2}\gamma \cdot TC_{SE}\cdot \Upsilon_{M,SE}\right)}^{-1}$, where $\Upsilon_{M,SE}\propto \left(1-\exp\left(-\frac{TR_{SE}}{T_{1}}\right)\right)\cdot \exp\left(-\frac{TE_{SE}}{T_{2}}\right)$. For *T_2_* and *B_z_* values in the ranges of 30–150 ms and 0–50 nT, ξ was computed by varying NES from 0 to 5, leading to contour plots of  ξ with respect to *T_2_* and *B_z_*. The simulation parameters in the ESSSI pulse sequence were kept identical to those above, while those in SE imaging were: TR = 1000 ms, TE = 40 ms, and TC = 32 ms.

### 2.4. Experimental Studies

Experimental studies were performed at 3 T (Siemens Trio, Erlangen, Germany) in three different custom-made cylindrical conductivity phantoms (hereafter referred to as phantoms A, B, and C). A 32-element head coil was used for signal reception. Phantoms A and B consisted of a homogeneous agar gel object with σ = 2.1 S/m (CuSO_4_, 0.5 g/L; NaCl, 10 g/L) but differed in their agar concentrations (A: 25 g/L; B: 10 g/L); thus, they simulated tissues with different $T_{2}^{*}$ values. Phantom C was composed of two cylindrical agar gels surrounded by saline solutions, in which the agar objects with larger and smaller diameters and the background solutions presented σ = 2.79 S/m (CuSO_4_, 1.25 g/L; NaCl, 12.5 g/L; agar, 25 g/L), σ = 1.14 S/m (CuSO_4_, 1.25 g/L; NaCl, 3.75 g/L; agar, 25 g/L), and σ = 0.2 S/m (CuSO_4_, 1 g/L; NaCl, 1.5 g/L), respectively. Prior to conductivity imaging, a multi-echo gradient echo scan (TR = 500 ms, flip angle = 60°, TEs = 2.2/4.0/6.2/8.4/10/6/15/20/30/50/80 ms) was performed to measure $T_{2}^{*}$ via a linear regression. The obtained $T_{2}^{*}\;$ maps served as a reference when experimentally validating the relationship between NES and *B*_z_ SNR simulated above.

Two sets of MREIT data were acquired with opposite polarities of the injection current, yielding a *B*_z_ map via Equation (10). In each measurement, a single line of the projection signal was collected without current injection, and then its phase was demodulated from current-encoded signals so as to correct for any systematic global phase offset. The above procedure was repeated with the direction of current injection rotated by 90°. Finally, given the two sets of *B*_z_ estimates (*B*_z,1_, *B*_z,2_) in the orthogonal directions, a conductivity map was constructed using the CoReHa software package [29], in which the harmonic *B*_z_ algorithm is implemented in a semi-automatic manner.

To investigate the effect of echo-shifting on $SD_{B_{z}}$ and for conductivity estimates, data were acquired in phantoms A and B using the proposed multi-slice interleaving ESSSI pulse sequence with increasing NES from 0 to 5 (increment: 1), leading to four sets of images—the magnitudes of S^+^ (|S^+^|), *B*_z,1_, *B*_z,2_, and σ. The magnitude SNR and $SD_{B_{z}}$ in seven regions-of-interest (ROIs) of the |S^+^| and *B*_z,1_ images, were estimated using the NEMA-I method [30]. To this end, two sets of images were obtained independently using the same imaging parameters, and the standard deviation of their difference in each ROI was computed, serving as a noise statistic. Given the $SD_{B_{z}}$ measurements, η was then calculated using Equation (12). Furthermore, standard deviations of σ (SD_σ_) in seven ROIs were measured and compared across the examined NES values. The imaging parameters were as follows: field-of-view = 180 × 180 mm^2^, slice thickness = 4 mm, number of slices = 6, in-plane matrix size = 128 × 128, readout bandwidth = 500 Hz/pix, TE = 5.5 ms, flip angle = 25°, number of signal averages = 2, and I = +10/−10 mA. For a given NES value, both TR (and thus T_r_) and TC were adjusted to minimum and maximum possible values, respectively, which were achievable with the above scan parameters: TR/TC = 50/6 ms (NES = 0, 1), 54/7 ms (NES = 2), 58/7.5 ms (NES = 3), 62/8 ms (NES = 4), and 68/9 ms (NES = 5).

Data were collected in phantom C using the proposed ESSSI MREIT method with increasing NES values from 0 to 3 (increment: 1), along with the conventional SE MREIT method for comparison. With both methods, three sets of images, |S^+^|, *B*_z,1_, and σ, were presented, respectively. Scanning parameters common to both imaging techniques were: field-of-view = 150 × 150 mm^2^, slice thickness = 4 mm, number of slices = 8, in-plane matrix size = 128 × 128, and I = +10/−10 mA. Parameters specific to the proposed method were: TR = 72 ms, TE = 4 ms, TC = 6 ms, flip angle = 15°, readout bandwidth = 500 Hz/pix, number of signal averages = 10, and scan time = approximately 6.5 min. The parameters specific to conventional SE MREIT were: TR = 1000 ms, TE = 40 ms, TC = 32 ms, number of signal averages = 3, and scan time = approximately 26 min. The experiments performed in this study and the relevant imaging parameters are summarized in Table 1.

## 3. Results

### 3.1. Numerical Simulations

**Figure 2:**
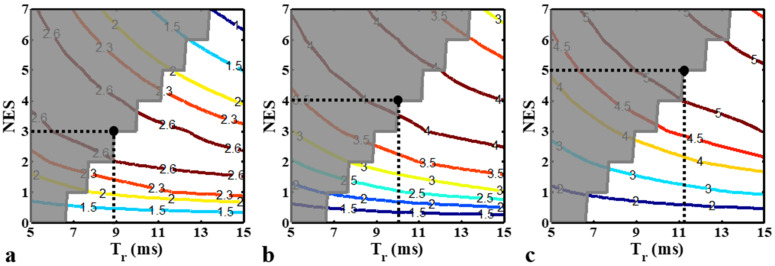
Contour plots of simulated η values for three different tissues with $T_{2}^{*}$ values of 40 (**a**), 70 (**b**), and 100 (**c**) ms with varying T_r_ and NES values. The solid circle with dotted lines indicates the contour level chosen in this study with the corresponding parameter values for each tissue. The region marked in dark gray represents parameter ranges that are challenging to use in practice due to hardware limits. Note that compared with the non-echo-shifting method (NES = 0), the proposed method with the selected parameters achieves *B_z_* SNR increases of 2.6 (**a**), 4.1 (**b**), and 5.2 (**c**)-fold for the three tissues, respectively.

Figure 2 shows contour plots of η with increasing T_r_ (5−15 ms) and NES (0–7) values for tissues with $T_{2}^{*}$ values of 40 ms (Figure 2a), 70 ms (Figure 2b), and 100 ms (Figure 2c), with practically demanding parameter combinations excluded. As both T_r_ and NES values rise, the η values for the three tissues increase up to a certain point and then decrease afterward. Furthermore, η changes more slowly with T_r_ than with small values of NES (0−2), while varying approximately equally in the two directions for large values of NES (≥3). As expected, tissues with higher $T_{2}^{*}$ values allow for a larger gain of *B_z_* SNR (Figure 2a vs. Figure 2c). With the chosen parameters of T_r_ and NES, the echo-shifting scheme, when compared with the non-echo-shifting (NES = 0), yields an increase of *B_z_* SNR by 2.6 (Figure 2a), 4.1 (Figure 2b), and 5.2 (Figure 2c) times for the three tissues, respectively.

**Figure 3:**
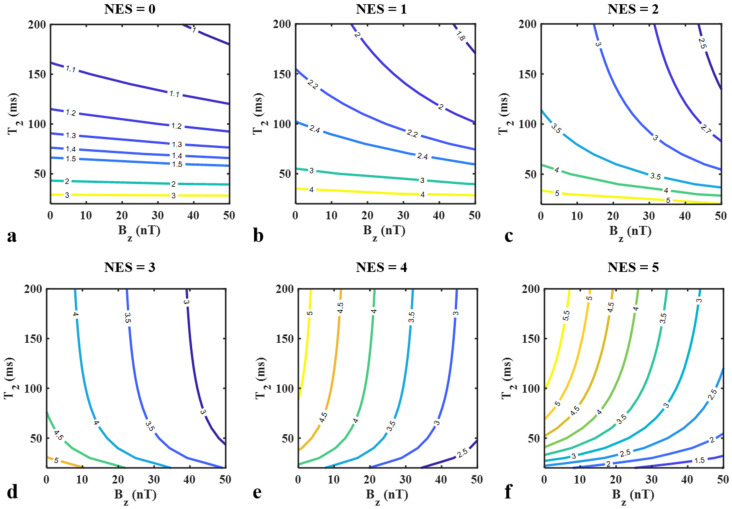
Contour plots of ξ for NES = 0 (**a**), NES = 1 (**b**), NES = 2 (**c**), NES = 3 (**d**), NES = 4 (**e**), and NES = 6 (**f**) in the proposed pulse sequence with varying *B_z_* (0–50 nT) and *T_2_* (20–200 ms) values. Note that the sensitivity of ξ to *B_z_* and *T_2_* changes with the NES values.

Figure 3 displays contour plots of ξ as a function of *B_z_* and *T_2_*, representing the relative *B_z_* SNR efficiency of the proposed method against conventional SE MREIT for NES = 0 (Figure 3a), NES = 1 (Figure 3b), NES = 2 (Figure 3c), NES = 3 (Figure 3d), NES = 4 (Figure 3e), and NES = 5 (Figure 3f). For all of the parameter combinations examined, ξ is larger than 1, thus suggesting that compared with the SE technique, the proposed ESSSI pulse sequence presents a substantially higher *B_z_* SNR efficiency. When a relatively smaller value of NES is employed (NES = 0–2), ξ decreases more rapidly with increasing *T_2_* more than it does with increasing *B_z_* (Figure 3a–c). In contrast, with moderately large NES values (NES = 3, 4), ξ changes predominantly toward the direction of *B_z_* (Figure 3d,e). Finally, when NES = 5, ξ increases with increasing *T_2_* and decreasing *B_z_* (Figure 3f).

### 3.2. Experimental Studies

**Figure 4:**
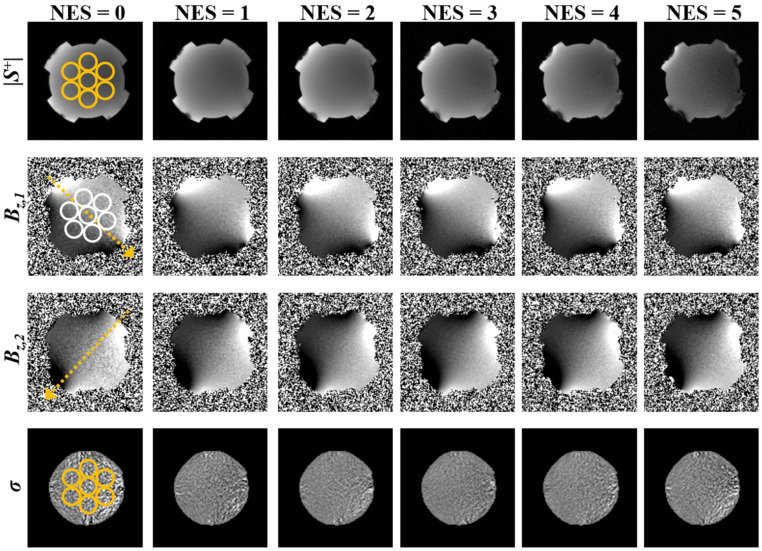
Four sets of images in phantom A ($T_{2}^{*}$ = 38 ms), |S^+^|, *B_z,1_*, *B_z,2_*, and σ in the row order, acquired using the ESSSI pulse sequence with varying NES values from 0 to 5 in the column order. The dotted arrows represent the direction of the injection current while the circles represent ROIs for statistical analysis.

**Figure 5:**
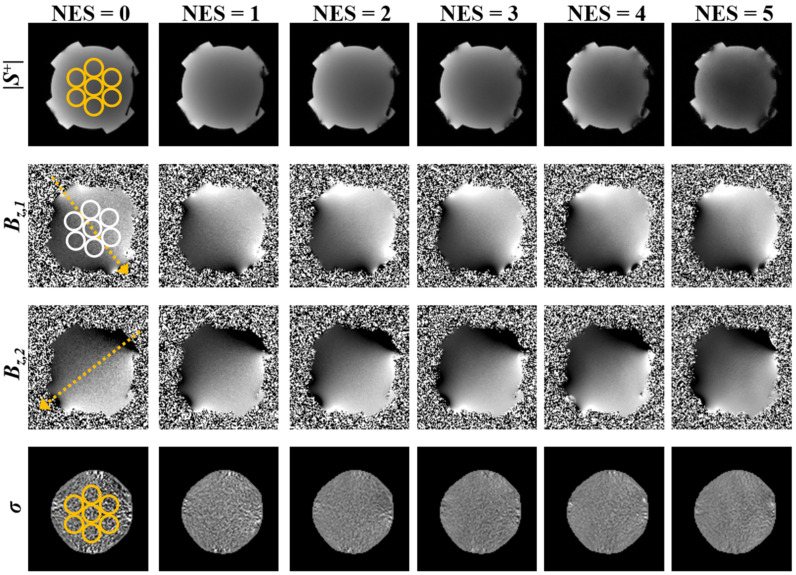
Images in phantom B ($T_{2}^{*}$ = 74 ms). Figure ordering is identical to that in Figure 4.

**Figure 6:**
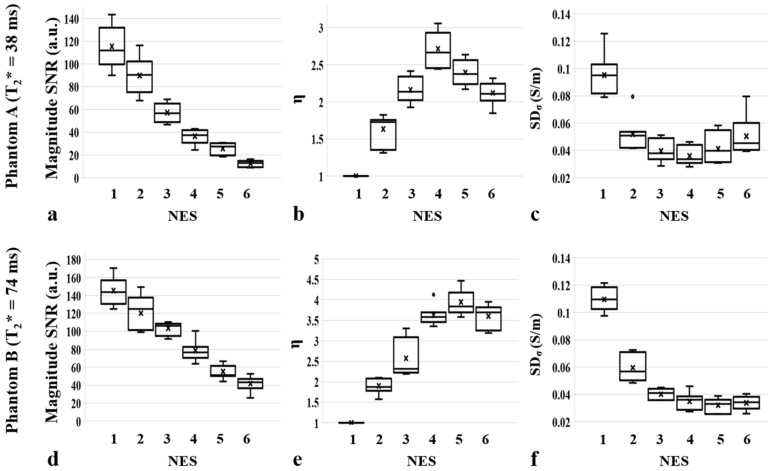
Boxplots of the magnitude SNR (**a,d**), η (**b,e**) and SD_σ_ (**c,f**) against NES for the selected ROIs in phantom A (**a**–**c**) and phantom B (**d**–**f**), respectively. Note that with increasing NES values, the magnitude SNR drops exponentially while η increases up to a certain value and then decreases afterwards in both phantoms. Correspondingly, SD_σ_ exhibits a reversed pattern to η. Note also that the behavior of η with respect to NES is closely matched with that shown in the numerical simulations (Figure 2a,b). In the boxplots, horizontal lines and x symbols represent median and mean values, respectively, while dots are outliers.

Figure 4 and Figure 5 show four sets of images (|S^+^|, *B_z,1_*, *B_z,2_*, σ) in phantom A and phantom B, obtained using the proposed ESSSI MREIT method with increasing NES values from 0 to 5 in the column order. The $T_{2}^{*}$ values of the agar gel objects were estimated as 38 ms (phantom A) and 74 ms (phantom B), respectively. Figure 6 plots the resultant boxplots of the magnitude SNR (Figure 6a,d), η (Figure 6b,e), and SD_σ_ (Figure 6c,f) values for the chosen ROIs in phantom A (Figure 6a–c) and phantom B (Figure 6d–f). As the NES values increase from 0 to 5, the magnitude image SNR drops approximately exponentially in both phantoms, while its decreasing rate in phantom A is relatively higher than that in phantom B (Figure 6a vs. Figure 6d). With increasing NES, η rises and falls at the NES values of 3 and 4 in phantom A (Figure 6b) and phantom B (Figure 6e), respectively, which experimentally validates the results of the numerical simulations (Figure 2a,b). As expected, SD_σ_ presents a reversed pattern in comparison to the variations of η against NES.

**Figure 7:**
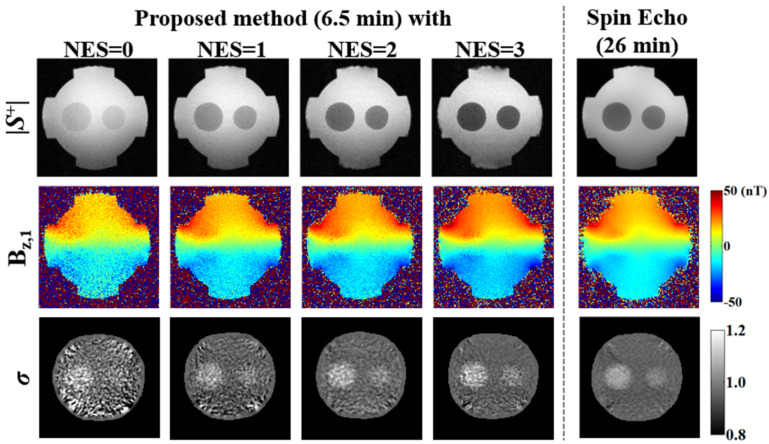
Comparison of images (phantom C) of |S^+^|, B_z,1_, and σ acquired using the proposed ESSSI MREIT with increasing NES values from 0 to 3 (first four columns) and the conventional SE MREIT method as the reference (last column).

Figure 7 compares three sets of images, |S^+^|, *B*_z,1_, and σ, in phantom C obtained using the ESSSI MREIT method with NES = 0–3 and conventional SE MREIT. As NES values increase from 0 to 3, the proposed method results in decreased signal intensity for the magnitude images but yields a gradual reduction in noise for the *B*_z_ estimates, leading to an elevated conductivity contrast. Furthermore, compared to the reference, namely the conventional SE MREIT method, the conductivity map obtained using the proposed method with NES = 3 depicts a similar level of conductivity contrast. Although the results for NES > 3 in the proposed method are not shown, the above results from numerical simulations (Figure 2) and homogeneous phantom experiments (Figure 4, Figure 5 and Figure 6) suggest that *B_z_* SNR and conductivity reconstruction would be degraded after a certain NES threshold.

## 4. Discussion

This work introduces a new MREIT method based on an echo-shifted steady-state incoherent imaging pulse sequence for rapid and current-efficient conductivity mapping without an apparent loss of measurement accuracy. In the proposed method, FID signals are shifted over one or more imaging slices and experience an accordingly increased number of injection current pulses. Therefore, the effective current duration is lengthened, leading to enhanced *B*_z_ SNR values relative to the non-echo-shifted counterpart. Nonetheless, the number of echo-shifts is limited by the $T_{2}^{*}$-related signal loss. Hence, the optimal NES value varies with the intrinsic relaxation of the tissues, as shown via numerical simulations (Figure 2 and Figure 3) and further validated by phantom experiments (Figure 4, Figure 5 and Figure 6).

In the original implementation of echo-shifted imaging [31] and its variants thereafter [32,33], FIDs were shifted over the direction of phase-encoding lines for the same imaging slice. Hence, in these early techniques, steady-state signals were weighted by ${\left(\cos\alpha /2\right)}^{2NES}$ and remained at a relatively low level if a high NES value was employed. In contrast, since echo-shifting in the present pulse sequence is integrated into the multi-slice interleaving data acquisition process, the FID signal for a given slice is unaffected by RF pulses for other imaging slices. Furthermore, the multi-slice interleaving configuration employs a long TR to accommodate multiple RF pulses, leading to an enhanced level of steady-state signals. Hence, compared with the original echo-shifted imaging technique, the proposed method substantially elevates the SNR of magnitude images and accordingly the *B*_z_ SNR.

As an alternative to the T_r_-periodic spoiler gradient pulses in the proposed method, echo-shifting with (NES + 1)T_r_-periodic spoilers can be considered [32], such that the size of spoiler A (M_A_; Figure 1) varies across the pulse train with a period of (NES + 1)T_r_, while that of spoiler B is set to zero (i.e., M_B_ = 0). For example, if NES = 1, M_A_ changes its sign alternately over T_r_ with its absolute moment held constant. Compared with the present implementation of the spoilers, the (NES + 1)T_r_-periodic approach shortens the minimum possible T_r_ and potentially enhances the scan efficiency. Nonetheless, with reduced T_r_, the duration for the current pulse needs to be decreased accordingly, leading to reduced *B*_z_ SNR. Additionally, in the presence of any uncompensated residual eddy currents, (NES + 1)T_r_-periodic spoilers make the signal phase vary periodically along the direction of phase encoding, potentially resulting in ghosting artifacts in the obtained images.

In the proposed MREIT method, two measurements need to be performed separately with opposite polarities of injecting current pulses to eliminate the background magnetic field in the estimation of current-induced *B*_z_. Instead, alternating current injection may be applied by continuously switching the polarities of current pulses across the entire pulse train to make the *B*_z_ estimation relatively more immune to global phase offset over scans, as well as potential magnetic field drifts during each measurement. However, alternating current injection in sync with an echo-shifted pulse sequence would be undesirable because current-induced phases are successively cancelled out along the pulse train if the echo-shifting mode is turned on.

## 5. Conclusions

In conclusion, it was demonstrated that a new echo-shifted steady-state incoherent imaging-based MREIT method enables rapid, high-resolution conductivity mapping. The echo-shifting strategy in combination with the multi-slice interleaving data acquisition approach allows efficient encoding of the current-induced magnetic field, thereby enhancing the SNR of *B*_z_ without an explicit increase in the number of current pulses or the current duration. It is expected that the proposed method will provide a novel and highly efficient way to measure conductivity information in MREIT studies.

## Figures and Tables

**Table 1:** Summary of experiments performed in this study and corresponding imaging parameters.

Imaging Parameters	Phantoms A and B (Homogeneous)	Phantom C (Non-Homogeneous)	
	Proposed Method	Proposed Method	Conventional SE
Field-of-view (mm^2^)	180 × 180	150 × 150	150 × 150
Slice thickness (mm)	4	4	4
Matrix size	128 × 128	128 × 128	128 × 128
Number of slices	6	8	8
TR (ms)	50–68	72	1000
TE (ms)	5.5	4	40
Flip angle (degree)	25	15	90 (excitation)/ 180 (refocusing)
Averages	2	10	3
Approximate scan time (min)	0.9–1.2	6.5	26
NES examined	0, 1, 2, 3, 4, 5	0, 1, 2, 3	-

## Data Availability

Data will be made available on request to the corresponding author.
